# Exosomes from tendon stem cells promote injury tendon healing through balancing synthesis and degradation of the tendon extracellular matrix

**DOI:** 10.1111/jcmm.14430

**Published:** 2019-05-31

**Authors:** Yunjiao Wang, Gang He, Yupeng Guo, Hong Tang, Youxing Shi, Xuting Bian, Min Zhu, Xia Kang, Mei Zhou, Jingtong Lyu, Mingyu Yang, Miduo Mu, Fan Lai, Kang Lu, Wan Chen, Binghua Zhou, Jiqiang Zhang, Kanglai Tang

**Affiliations:** ^1^ State Key Laboratory of Trauma, Burn and Combined Injury, Department of Orthopeadics/Sports Medicine Center Southwest Hospital, Third Military Medical University Chongqing China; ^2^ Department of Neurology Third Military Medical University Chongqing China

**Keywords:** exosomes, tendinopathy, tendon stem cells

## Abstract

Tendon injuries are common musculoskeletal system disorders in clinical, but the regeneration ability of tendon is limited. Tendon stem cells (TSCs) have shown promising effect on tissue engineering and been used for the treatment of tendon injury. Exosomes that serve as genetic information carriers have been implicated in many diseases and physiological processes, but effect of exosomes from TSCs on tendon injury repair is unclear. The aim of this study is to make clear that the effect of exosomes from TSCs on tendon injury healing. Exosomes were harvested from conditioned culture media of TSCs by a sequential centrifugation process. Rat Achilles tendon tendinopathy model was established by collagenase‐I injection. This was followed by intra‐Achilles‐tendon injection with TSCs or exosomes. Tendon healing and matrix degradation were evaluated by histology analysis and biomechanical test at the post‐injury 5 weeks. In vitro, TSCs treated with interleukin 1 beta were added by conditioned medium including exosomes or not, or by exosomes or not. Tendon matrix related markers and tenogenesis related markers were measured by immunostaining and western blot. We found that TSCs injection and exosomes injection significantly decreased matrix metalloproteinases (MMP)‐3 expression, increased expression of tissue inhibitor of metalloproteinase‐3 (TIMP‐3) and Col‐1a1, and increased biomechanical properties of the ultimate stress and maximum loading. In vitro, conditioned medium with exosomes and exosomes also significantly decreased MMP‐3, and increased expression of tenomodulin, Col‐1a1 and TIMP‐3. Exosomes from TSCs could be an ideal therapeutic strategy in tendon injury healing for its balancing tendon extracellular matrix and promoting the tenogenesis of TSCs.

## 1 INTRODUCTION

Tendon is highly prone to injury during sports and other rigorous physical activities.^1^ Tendinopathy is a common chronic musculoskeletal system disorders worldwide, which exerts a significant detrimental effect on the patients' life quality. Many treatments for tendinopathy have been applied in clinical practice to restore tendon function and maintain the patient's quality of life, including non‐steroid anti‐inflammation drugs (NSAIDs) intake, administration of steroid injections and physical therapies.^2^, ^3^ However, their effects are largely limited to pain control and tendon matrix reconstruction. Tendon stem cells (TSCs) were first isolated from human and mouse in 2007 and were confirmed subsequently in rat and rabbit tendons.^4^, ^5^, ^6^ Tendon stem cells has characters of stem cell like self‐renewal ability, multi‐differentiation potential, colony formation ability, which enables them to differentiate into tenocytes, adipocytes, chondrocytes and osteocytes, and they have been widely used in tissue engineering and tendon healing.

Exosomes are small membrane vesicles of endocytic origin that are secreted by most cells in culture. As one of the major pathways of extracellular signalling, exosomes have been implicated in many diseases and participated in many physiological processes.^7^, ^8^, ^9^, ^10^ These are derived from fusion in cell to cell communication, as well as carriers of genetic information, which play an important role in inflammation and tissue repair when shuttled by exosomes.^11^, ^12^, ^13^ Reports have showed that exosomes from mesenchymal stem cells (MSCs) become potential regenerative medicine application for tendinopathy.^14^ Tendon stem cells are as a kind of MSCs, but effects of exosomes derived from TSCs on tendinopathy is unclear.

In this study, we reported that exosomes from TSCs balanced the matrix synthesis and degradation of the injury tendon and promoted the injury tendon healing. Exosomes from TSCs could be an ideal therapeutic strategy in tendon injury healing for its balancing tendon extracellular matrix and promoting the tenogenesis of TSCs.

## 2 MATERIALS AND METHODS

### 2.1 Ethics statement

Eight‐week‐old Sprague Dawley rats weighting 200‐250 g were used and housed under a 12 hour light/dark cycle in a pathogen‐free area with free access to water and food. All animals were treated according to institutional guidelines for laboratory animal treatment and care. All experimental procedures were approved by the Animal Research Ethics Committee of the Third Military Medical University, China.

### 2.2 Animal model establishment

A total of 18 male Sprague‐Dawley rats (8 weeks old, 200‐250 g) were divided into three groups (six/group): control group, injury group with TSCs treatment and injury group with exosomes treatment. First, in the two injury groups, rats were injected with 30 µL type I collagenase solution (10 mg/mL) into both Achilles tendons to establish micro‐damaged Achilles tendon model. After 1 week, rats in the injury group with TSCs treatment were injected by TSCs into the left injury site (PBS/right side), and rats in the injury group with exosomes treatment were injected by exosomes (twice a week) into the left injury site (PBS/right side). After 4 weeks, the tendon samples were collected for the next studies.

### 2.3 Histomorphometry

Achilles tendon specimens were fixed in 4% paraformaldehyde. Nine sections were cut at a thickness of 4 mm and stained with haematoxylin and eosin. To analyse the changes of total histological scores on haematoxylin and eosin‐stained slides after treatment, we used the established histological scoring system by Stoll^15^ et al. The score of the intact group was defined as 20 points.

### 2.4 Isolation and identification of exosomes

**Figure 1:**
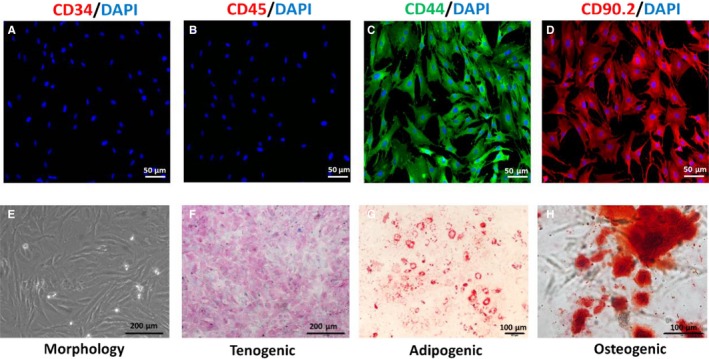
Establishment and identification of tendon stem cells (TSCs). (A‐D) Specific molecular profile of the TSCs. Expression of CD3 and CD34 were negative, but CD44 and CD90 expression were positive. E, Morphology of TSCs. (F‐H) Identification of tenogenic, adipogenic and osteogenic differentiation of TSCs, which were staining by Sirius red, Oil Red O and Alizarin red respectively

The isolation followed the multistep ultracentrifugation process as previously described.^16^ We finally isolated 1 mL exosomes (resuspended in PBS) from 1000 mL of TSCs culture medium. When the fusion rate of TSCs reached 70%‐80%, we changed the medium as the medium without foetal bovine serum (FBS). After 24 hours, the TSCs culture medium were ultilized for the isolation of exosomes. In our study, conditioned medium with exosomes (CM + Exo), conditioned medium without exosomes (CM − Exo) and exosomes were collected for treating TSCs (Figure 1A). Identification of exosomes was through transmission electron microscope (TEM), particle diameter analysis and Western blotting for specific markers.

### 2.5 Isolation, identification and culture of rat TSCs

Isolation of rat TSCs were performed as our previously described.^14^ Briefly, Achilles tendons from both hind feet were dissected after euthanasia. Only the mid‐substance tissue was collected, and peritendinous connective tissue was carefully removed. Mid‐substance tissue was minced in sterile PBS and digested in 3 mg/mL of type I collagenase (Sigma‐Aldrich, St. Louis, MO), 2.5 hours at 37°C. A 70 mm cell strainer (Becton Dickinson, Franklin Lakes, NJ) was used to yield a single‐cell suspension. The released cells were washed in PBS and centrifuged at 300 *g* for 5 minutes, then resuspended in DMEM (Gibco, Carlsbad, CA) with 10% FBS, 100 U/mL penicillin, 100 mg/mL streptomycin and 2 mmol/L L‐glutamine (all from Invitrogen, Carlsbad, CA). Increasing dilutions of the isolated cells were plated and grown for 2 days at 37°C in 5% CO_2_, then washed twice in PBS to remove non‐adherent cells. On day 7 of culture, the cells were trypsinized with trypsin‐ethylenediaminetetraacetic acid (EDTA) solution (Sigma‐Aldrich), mixed together and cultured as passage 0 cells. Cells from passages 3 (P3) were used in the subsequent experiments. For identification of TSCs, specific markers of CD34 (1:200; Abcam), CD44 (1:200; Abcam), CD45 (1:200; Abcam) and CD90 (1:200; Abcam) were tested through immunostaining analysis. Tendon stem cells were seeded onto six‐well plates for real‐time quantitative PCR (qRT‐PCR) and scratch assays, and 10‐cm‐diameter petri dishes for protein extraction.

For CM ± Exo treated analysis, after seeding for 24 hours, we changed the two wells of four as CM − Exo and CM + Exo. After that, we added with or without 10 ng/mL interleukin 1 beta (IL‐1β; PeproTech, Rocky Hill, NJ). For exosomes treated analysis, after seeding for 24 hours, 10 ng/mL IL‐1β were added into TSCs for 1 hour, and then exosomes was added. After 48 hours, TSCs were collected for the next study.

#### 2.5.1 Tri‐lineage differentiation assay

Multidifferentiation potential of TSCs was tested under tenogenic, adipogenic and osteogenic induction according to previously described.^17^ Briefly, tenogenic differentiation of TSCs was induced with low glucose DMEM (LG‐DMEM) supplemented with ascorbic acid (25 μmol/L) (Sigma, USA) and connective tissue growth factor (CTGF; 25 ng/mL) (Human CTGF; PeproTech). Adipogenic and osteogenic induction medium were purchased (Cyagen, Suzhou). The medium was changed every 3 days. After 2 weeks, TSCs were assessed by Sirius red staining, oil red staining and Alizarin red staining.

### 2.6 Protein extraction and Western blotting

The cells were washed twice with PBS and lysed in lysis buffer (50 mmol/L Tris‐HCl, pH 8.0, 1 mmol/L EDTA, 1% Triton X‐100, 0.5% sodium deoxycholate, 0.1% sodium dodecyl sulfate [SDS], 150 mmol/L NaCl) containing a mixture of proteinase inhibitors (Thermo Fisher Scientific Inc, Rockford, IL). Total protein concentrations were measured using a bicinchoninic acid protein assay kit (Thermo Fisher Scientific Inc), and equal amounts of proteins samples (30 µg/lane) were resolved by SDS‐PAGE and then transferred onto polyvinylidene difluoride membranes, and membranes blocked by incubating with 5% non‐fat milk containing 0.1% tris Buffered saline Tween (TBST) for 2 hours at room temperature. The membranes were then incubated sequentially with primary antibodies overnight at 4°C. The following primary antibodies were used: anti‐Col‐1 (1:2000; Abcam), anti‐TNMD (1:2000; Abcam), anti‐matrix metalloproteinase (MMP)‐3 (1:2000; Cell Signalling Technology), anti‐TIMP‐3 (1:2000; Cell Signaling Technology). Glyceraldehyde‐3‐phosphate dehydrogenase (GAPDH) (1:5000; Proteintech) was used as an internal control. Following primary antibody incubation, membranes were washed three times in 0.1% TBST and incubated in goat anti‐rabbit IgG (H&L)‐horseradish peroxidase conjugate (1:2000; Proteintech) 2 hours at room temperature. Proteins were visualized and images captured using a LiCor Odyssey Imager (LI‐COR Biosciences, Lincoln, NE).

#### 2.6.1 Immunostaining

Serial coronal frozen sections (5 μm thick) were prepared from Achilles tendons as previously described.^1^ Briefly, sections were rewarmed at room temperature for 30 minutes and washed in PBS for four times every 5 minutes. With punching and blocking the sections with 0.1% Triton 100 and 5% bovine serum albumin for 1 hour, sections were incubated with matrix metabolism related markers Col‐1 (1:200; Abcam), MMP (1:200; Cell Signaling Technology) and tissue inhibitor of metalloproteinase‐3 (TIMP‐3) (1:200; Cell Signaling Technology) at 4°C overnight. Sections were incubated with secondary antibodies (1:200; Proteintech) for 20 minutes. Treated with goat anti‐rabbit IgG H&L (Dylight‐594) (1:200; Proteintech) for 1 hour. After washed for four times every 5 minutes, sections were counterstaining with (4′,6‐diamidino‐2‐phenylindole, DAPI) for 10 minutes. At last, sections were mounted by fluorescence quencher.

### 2.7 Biomechanical analysis

We followed the procedures as described in previous study.^18^ The Achilles's tendon with bony end was first isolated. The two bony ends of tendon were fixed on a custom‐made testing jig with two clamps. The lower one was used to fix the calcaneus end while the upper one was used to fix the tibia end. The whole construct was then mounted onto the mechanical testing machine.

### 2.8 Statistical analysis

All values were expressed as mean ± standard deviation (SD). The Student's *t* test was used to compare between two groups. Multiple comparisons were made using a one‐way ANOVA followed by Fisher's tests. A *P*‐value of <0.05 was considered to be statistically significant.

## 3 RESULTS

### 3.1 Identification of TSCs

First, we used immunostaining to examine the specific surface antigens on TSCs (Figure 1A‐D). The results showed that TSCs were positive for the fibroblast marker CD90 that was not expressed by bone marrow MSCs (BMSCs). They were negative for the haematopoietic stem cell markers CD34 and for the leukocyte marker CD45. Tendon stem cells were positive for BMSCs markers CD44. The results showed that TSC was a kind of BMSCs but not BMSCs. Then, we observed the morphology of TSCs. At low density, TSCs showed spindle‐shaped and fibroblast‐like morphology. When TSCs fused to 80%‐90%, TSCs showed pebble‐like morphology and developed into tight colonies (Figure 1E). Upon tenogenic induction, TSCs were stained with Modena by Sirius red, showing collagen gathering (Figure 1F). After adipogenic induction, these cells displayed round orange droplets within the cytoplasm upon oil red staining, which indicates formation of lipid droplets (Figure 1G). Under osteogenic induction, TSCs synthesized matrix which were stained dark brown by Alizarin red, indicating calcium deposition (Figure 1H).

### 3.2 Isolation and identification of exosomes from TSCs

**Figure 2:**
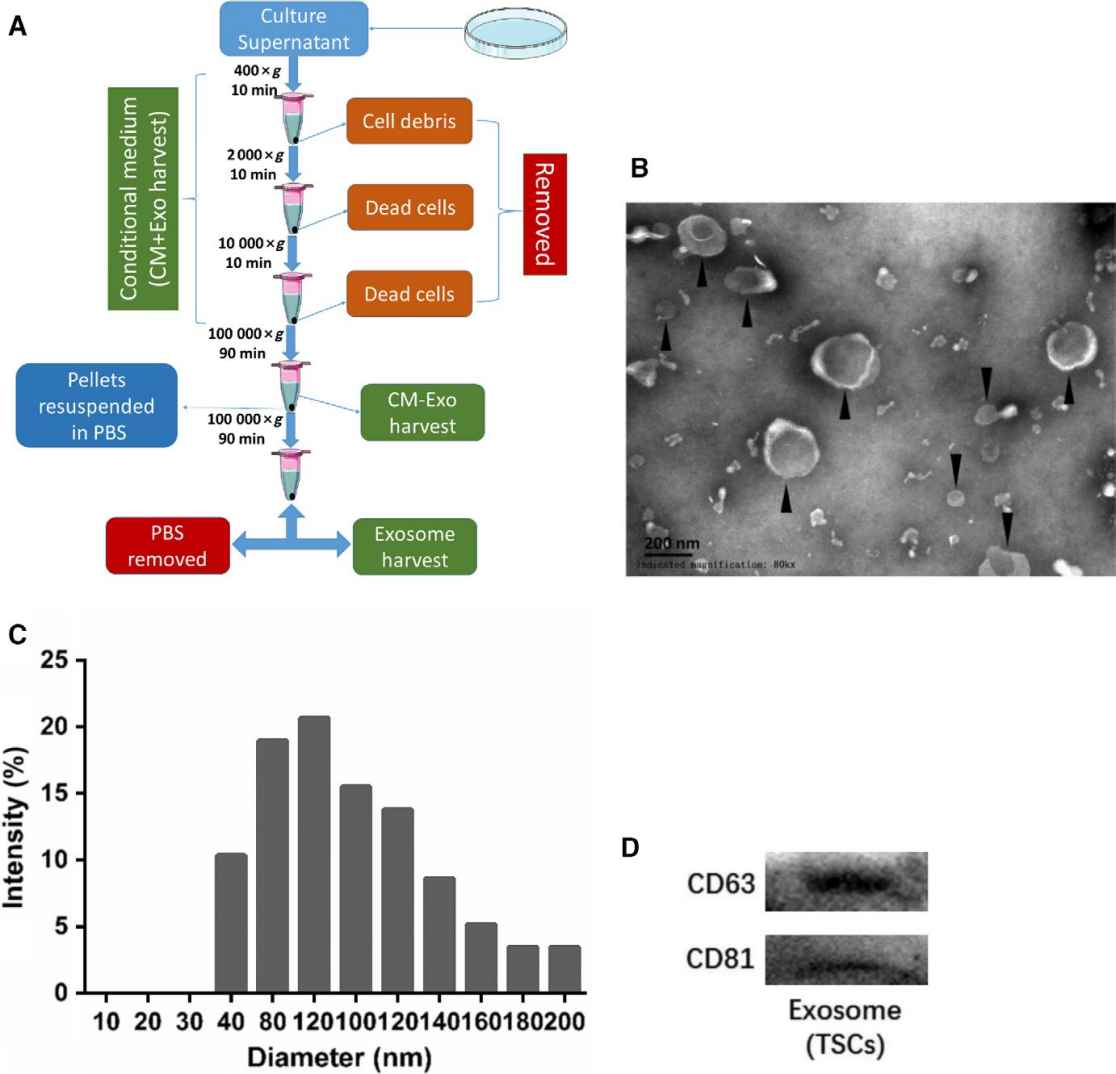
Isolation and identification of exosomes derived from tendon stem cells (TSCs). A, Flowchart of exosomes, conditioned medium and conditioned medium without exosomes preparation. B, Exosomes observed under transmission electron microscope. Scale bar, 200 nm. C, Diameter distribution of isolated exosomes. D, Expression of specific markers of CD63 and CD81 by exosomes derived from TSCs

To clarify whether exosomes or cytokines from TSCs have influence on the alleviation of tendinopathy, CM + Exo, CM − Exo and exosomes from TSCs culture supernatant were harvest through ultra‐high speed gradient centrifugation for next analysis (Figure 2A). The concentration of the isolated exosomes is 486.3 μg/mL. Under the TEM, exosomes appeared round or oval vesicles with lipid bilayers (Figure 2B). Size distribution by intensity showed exosomes from TSCs were approximately 40‐200 nm in diameter (Figure 2C). Results of Western blotting confirmed that vesicles expressed specific markers of CD63 and CD81 (Figure 2D).

### 3.3 TSCs injection promoted the injury tendon healing

**Figure 3:**
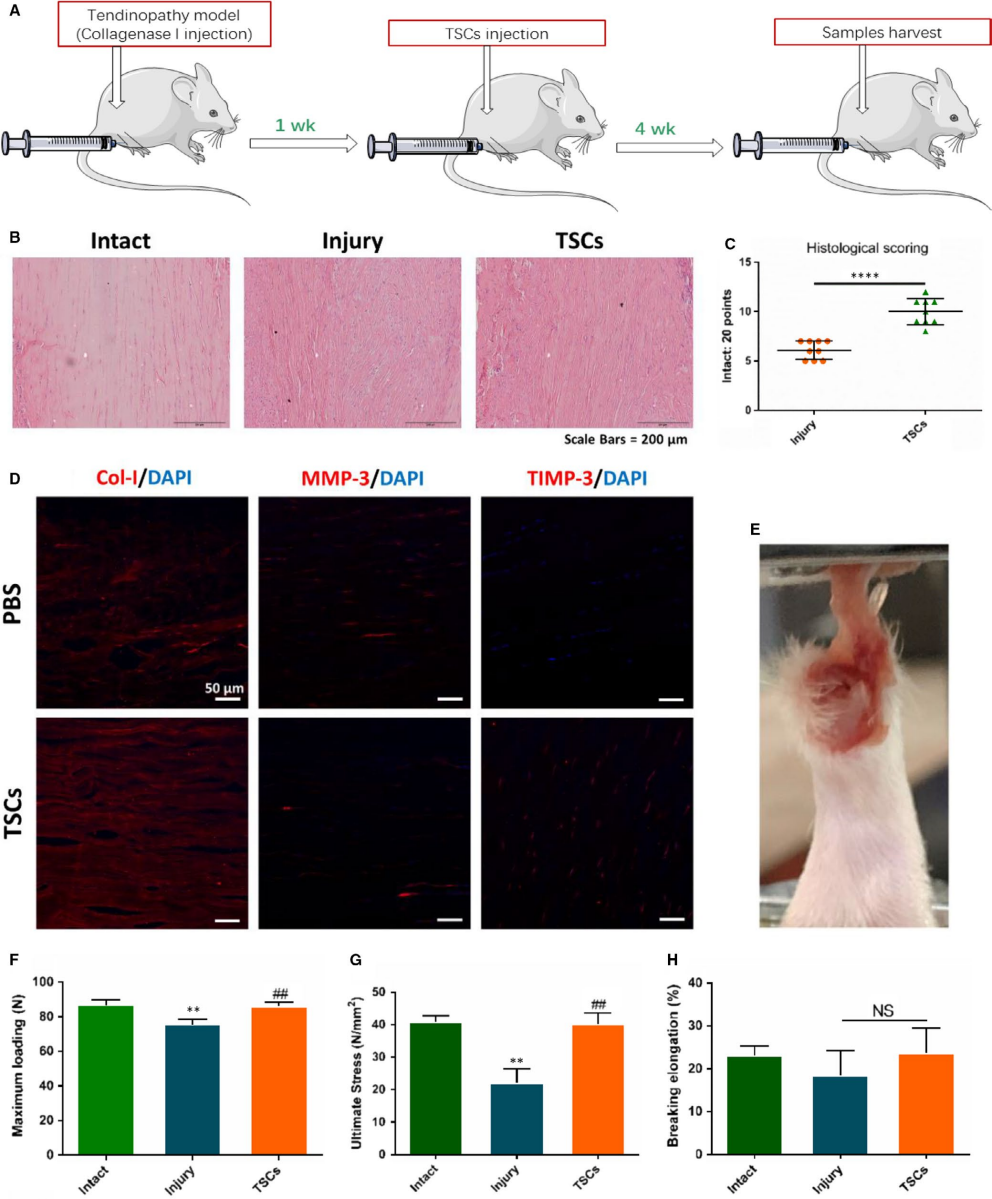
Effects of tendon stem cells (TSCs) injection on injury tendon healing on the tendinopathy model. A, Flowchart of tendinopathy establishment, TSCs injection and samples collected. B, Haematoxylin and eosin staining showed the tendon healing status of intact, injury and TSCs treatment group. C, Histological score for injury and TSCs treatment tendon, N = 9. D, Immunostaining showed the level of Col‐1, matrix metalloproteinases (MMP)‐3 and tissue inhibitor of metalloproteinase‐3 (TIMP‐3) between injury and TSCs treatment group. E, Biomechanical test was performed. (F‐H) Biomechanical properties was measured. *P < 0.05, **P < 0.01, ^#^:vs Injury group, NS, no significance, N = 6

To evaluate the therapeutic effects of TSCs on tendinopathy, we injected 20 µL of cell suspension (1 × 10^6^) directly in the left Achilles tendon and the left side was injected with 20 µL of PBS at 1 week after 30 µL of collagenase I injection in the bilateral Achilles tendon to establish tendinopathy model. We harvested the tendon samples after following 4 weeks (Figure 3A). Haematoxylin and eosin staining showed that collagen fibers in TSCs treatment group had a more ordered arrangement than PBS group (Figure 3B). Histological score system also showed that score of TSCs group was significantly higher than that of PBS injury group (*P* < 0.0001). The results revealed that TSCs injection had the therapeutic effect on injury tendon healing (Figure 3C). Immunohistochemistry and biomechanical test also verified the results above. Tendon stem cells group exhibited much stronger extracellular matrix (ECM) related markers of Col‐I and TIMP‐3, and weaker markers of MMP‐3(Figure 3D). Maximum loading and ultimate stress in TSCs group were lifted significantly compared with injury group (*P* = 0.0041 and *P* = 0.0108; Figure 3E‐H).

### 3.4 TSCs culture medium deficient in exosomes promoted the tendon matrix maintenance in vitro

**Figure 4:**
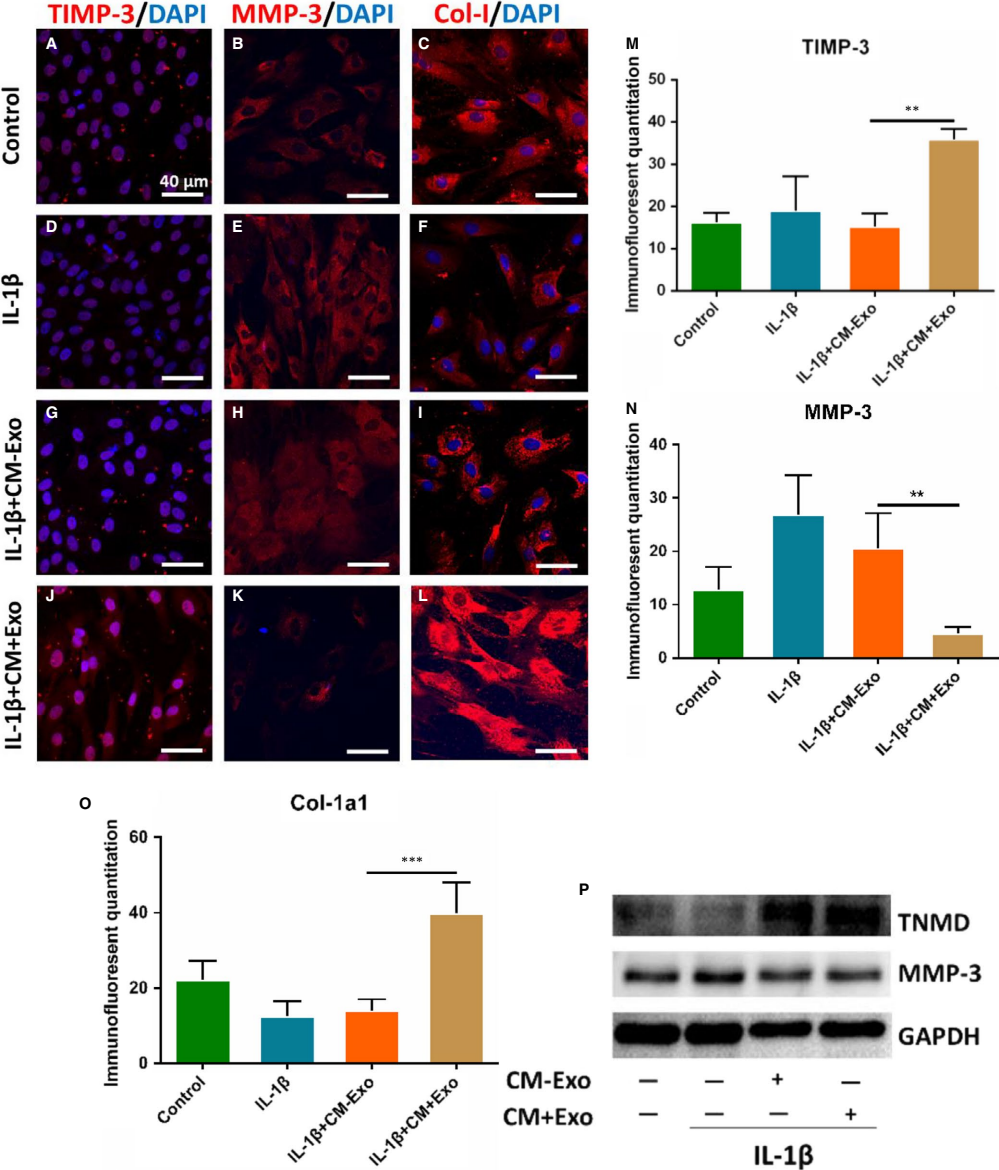
Effects of TSCs culture medium deficient in exosomes on the tendon matrix maintenance in vitro. TSCs were treated by IL‐1β, IL‐1β + CM + Exo and IL‐1β + CM−Exo. (A‐O) Immunostaining showed the expression level of Col‐1, MMP‐3 and tissue inhibitor of metalloproteinase‐3 (TIMP‐3) among the four groups. P, Western blotting showed protein expression of tenomodulin (TNMD) and MMP‐3. Glyceraldehyde‐3‐phosphate dehydrogenase (GAPDH) was used as the internal. *P < 0.05, **P < 0.01, N = 3. CM + Exo, conditioned medium with exosomes; CM−Exo, conditioned medium without exosomes; IL‐1β, interleukin 1 beta; MMP, matrix metalloproteinases; TSC, tendon stem cell

To make clear that whether exosomes or cytokines exert the effect on balancing injury tendon matrix by TSCs, we harvested the CM + Exo and the CM − Exo. First, we added IL‐1β into TSCs to establish inflammation model for 0.5 hour in vitro. Then, we added the CM + Exo and the CM − Exo respectively. Immunofluorescence showed that IL‐1β significantly promoted the synthesis of MMP‐3 (*P* = 0.0052) and increased the expression of Col‐1 and TIMP‐3 (*P* = 0.0009 and *P* = 0.0019). Conditioned medium with exosomes could reversed this effect but not CM − Exo (Figure 4A‐O). Western blotting showed that expression of tenomodulin (TNMD) was obviously promoted and MMP‐3 was inhibited in CM + Exo group (Figure 4P).

### 3.5 Exosomes derived from TSCs promoted the tendon matrix maintenance in vitro

**Figure 5:**
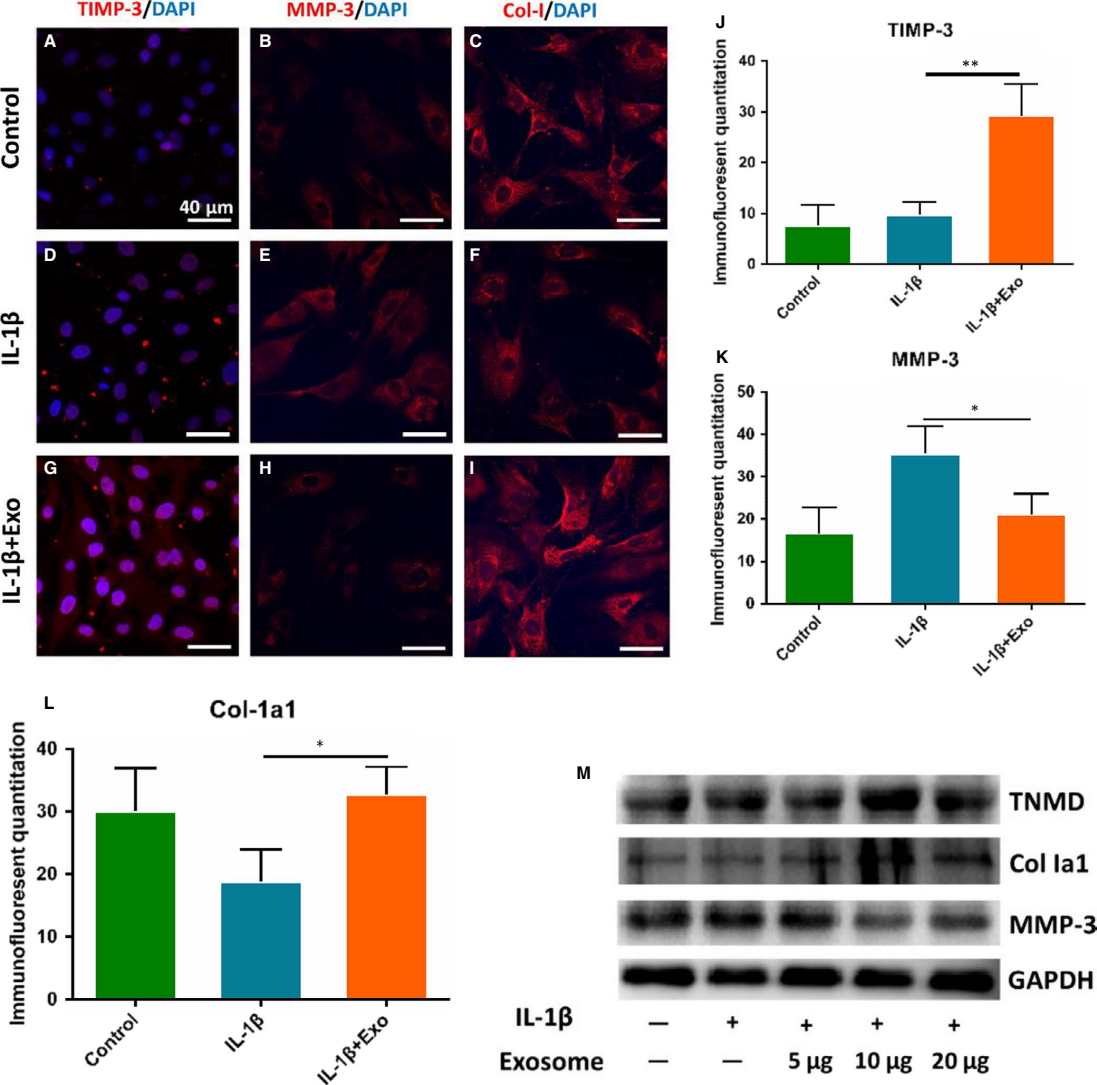
Effects of exosomes derived from tendon stem cells (TSCs) on the tendon matrix maintenance. TSCs were treated by interleukin (IL)‐1β and exosomes. (A‐I) Immunostaining showed the expression level of Col‐1, matrix metalloproteinases (MMP)‐3 and tissue inhibitor of metalloproteinase‐3 (TIMP‐3) among the three groups. M, Western blotting showed protein expression of tenomodulin (TNMD), Col‐1, MMP‐3 and TIMP‐3. Glyceraldehyde‐3‐phosphate dehydrogenase (GAPDH) was used as the internal. *P < 0.05, **P < 0.01, N = 3

Next, we evaluated the effect of exosomes derived from TSCs on tendon matrix maintenance through immunostaining analysis and Western blotting. We added exosomes into TSCs before IL‐1β pretreatment for 0.5 hour. Immunostaining showed that MMP3 expression in the exosomes treated group significantly decreased(*P* = 0.0182), and the TIMP3 and Col‐1a1 expression in this group significantly increased(*P* = 0.0020 and *P* = 0.0470) (Figure 5A‐L). Meanwhile, Western blotting showed that TNMD and Col‐1a1 expression increased and MMP3 expression decreased after exosomes' adding (Figure 5M).

### 3.6 Exosomes derived from TSCs promoted injury tendon healing

**Figure 6:**
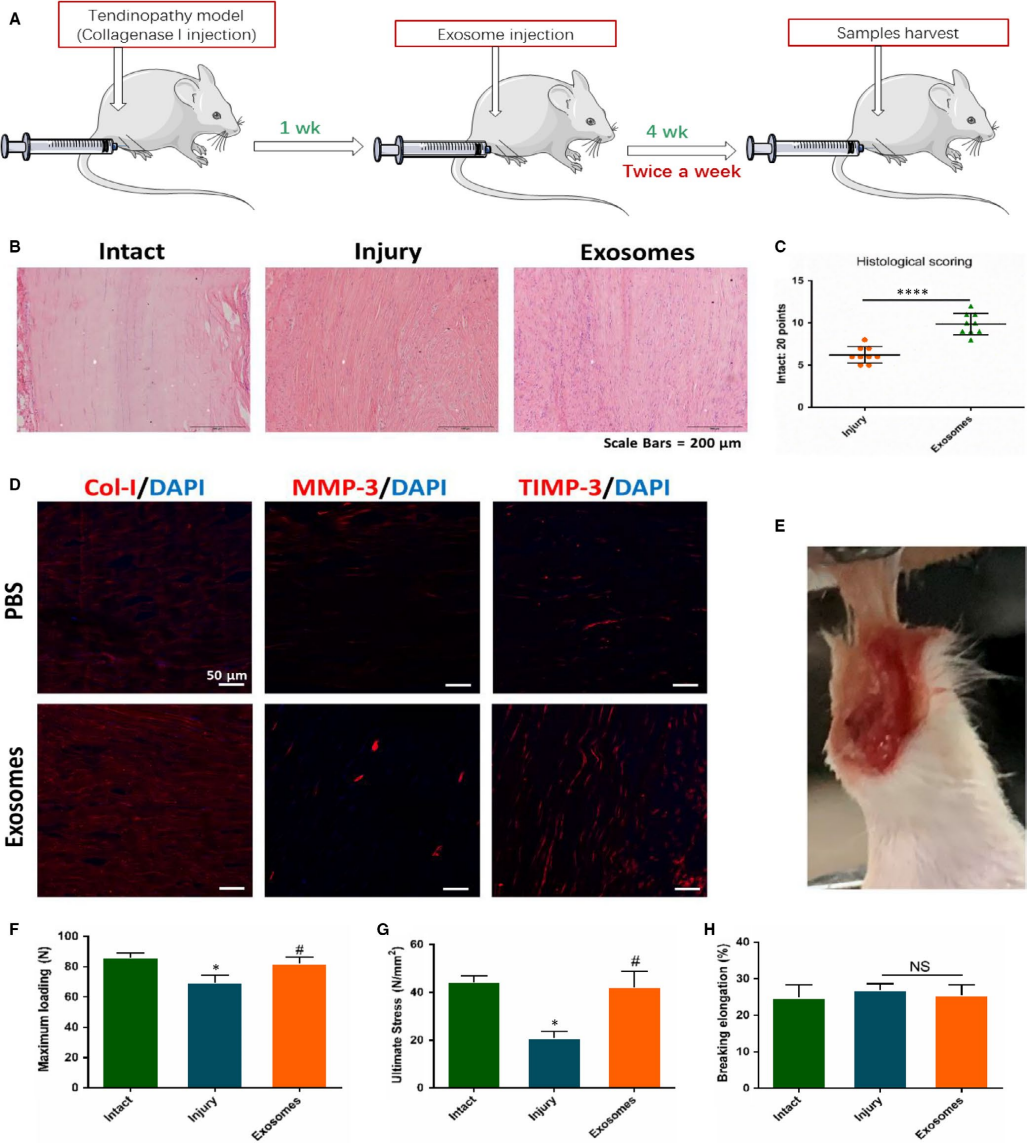
Effect of exosomes derived from TSCs on the injury tendon healing. A, Flowchart of tendinopathy establishment, exosomes injection and samples collected. B, Haematoxylin and eosin staining showed the tendon healing status of intact, injury and exosomes treatment group. C, Histological score for injury and exosomes treatment tendon, N = 9. D, Immunostaining showed the level of Col‐1, matrix metalloproteinases (MMP)‐3 and tissue inhibitor of metalloproteinase‐3 (TIMP‐3) between injury and exosomes treatment group. E, Biomechanical test was performed. (F‐H) Biomechanical properties was measured. *P < 0.05, ****P = 0.000, ^#^: vs Injury group, NS, no significance, N = 6

At last, we observed the effect of the exosomes derived from TSCs on alleviation of tendinopathy (Figure 6A). From the haematoxylin and eosin staining, we found that the arrangement of collagens in exosomes group were more uniform than that of injury group (Figure 6B). The quantification results of histological score showed that exosomes treatment significantly increased the score after tendon injury (*P* < 0.0001; Figure 6C). Immunostaining results also revealed that MMP3 was lowered and TIMP3 and Col‐Ia1 were elevated by exosomes, which was in accordance with that in vitro (Figure 6D). Finally, we conducted the biomechanical analysis test, which showed that maximum loading and ultimate stress in exosomes group were significantly higher than that in injury group (*P* = 0.0117 and *P* = 0.0112; Figure 6E‐H).

## 4 DISCUSSION

To restore structure and function of injury tendon is a big challenge in sports medicine because the understanding of tendon biology, and the molecular mechanisms controlling the migration, proliferation and fate of TSCs during tendon repair are lacking. Generally, the process of tendon healing includes three phases.^19^ First, inflammatory cells especially neutrophils are gathered to the injury site, and collagen‐III synthesis is initiated. Then, proliferative phase begins, and after 6 weeks, the remodelling phase commences. Although tendon has the ability of selfheal through the process above, the healed tissue never match the biochemical and mechanical properties of intact tendon.^20^

It is well reported that the TSCs and their cytokines executed many therapeutic effects on tendon injury healing process. Solaiman Tarafder et al^21^ showed that tendon stem/progenitor cells regulate inflammation in tendon healing via c‐Jun N‐terminal kinase and signal transducer and activator of transcription 3 signalling. Zhijin Yang et al^22^ reported that effect of TSCs in chitosan/β‐glycerophosphate/collagen hydrogel on Achilles tendon healing in a rat model. Jianying Zhang et al^23^ found that the role of engineered tendon matrix in the stemness of TSCs in vitro and the promotion of tendon‐like tissue formation in vivo, and indicated that engineered tendon matrix may be used to effectively expand TSCs in vitro and with TSCs, to enhance repair of injured tendons in vivo. Chunlai Tan et al^24^ demonstrated that transplantation of *Scx*‐transduced tendon‐derived stem cells (TDSCs) promoted better tendon repair compared to the transplantation of mock‐transduced cells. Our study showed that TSCs treatment improved the injury tendon healing and biomechanical properties. The results above gave us basic evidence for the next exploration.

Exosomes, as one of the major pathways of extracellular signalling, have been implicated in many diseases and participate in many physiological processes. They carry the genetic materials and execute the effect on cells through cell to cell fusion and communication. Haomin Cui et al^25^ reported that exosomes containing miRNA derived from macrophages induce peritendinous fibrosis healing after tendon injury. Yafei Wang and S. Zhang et al^26^, ^27^ revealed that exosomes from embryonic MSCs alleviate osteoarthritis through balancing synthesis and degradation of cartilage extracellular matrix. Myeongsik Oh et al^28^ illustrated that exosomes derived from human induced pluripotent stem cells ameliorate the aging of skin fibroblasts. Up to now, there is no related reports about the effect of exosomes derived from TSCs on balancing synthesis and degradation of injury tendon and tendon healing process. In this study, we successfully isolated and identified exosomes from TSCs and firstly demonstrated that exosomes from TSCs alleviated tendinopathy and improved the biomechanical properties. Results above illustrated that exosomes derived from TSCs played a vital role in tendon injury healing process and balancing tendon microenvironment and matrix.

Matrix metalloproteinases are a large family of zincdependent endopeptidases that are crucial to ECM remodelling.^29^ Matrix metalloproteinases function in the modification of essentially all components of the ECM, including collagens, proteoglycans and fibronectins. They are also involved in modulating the activity of signalling molecules and play important roles in both normal physiology and pathological processes.^30^, ^31^, ^32^ TIMPs, which are inhibitors of MMPs, have been hypothesized to account for the impaired healing ability.^33^, ^34^, ^35^, ^36^ Matrix metalloproteinase activity is regulated by the formation of complexes with TIMP‐1, ‐2, ‐3, ‐4. The inhibitory effects of the TIMPs are overlapping, since they are not specific to a single MMP group.^37^ TIMPs are able to interfere with the active, as well as latent, form of MMPs and in addition to their inhibitory activities they also have promoting functions. TIMPs play an important role in the regulation of cell growth, differentiation, apoptosis and angiogenesis.^38^, ^39^ Based on this, a balance between matrix MMPs and their inhibitors TIMPs is required to maintain tendon homeostasis.^19^ Variation in this balance over time might impact on the success of tendon healing.^18^ Hence, balance between MMPs and TIMPs are important potential targets for controlling such processes and in treating of a variety of pathological conditions. At the same time, we detected the expression of Col‐1a1 and TNMD, the results showed that the two tenogenic markers were elevated by exosomes. To this point, exosomes also have promotion effects on tenogenic healing process, which in turn increased injury tendon healing quality and biomechanical properties.

In summary, this study demonstrated that exosomes derived from TSCs relieved the expression of MMP3 and elevated expression of TIMP3, Col‐1a1 and TNMD, which balanced and maintained the homoeostasis of tendon. At the same time, the biomechanical properties of the injury tendon were improved by exosomes. The results above providing a new target for tendon injury drug and drug‐delivery system development.

## CONFLICT OF INTEREST

No competing financial interests exist among any authors in relation to this submission.

## AUTHOR CONTRIBUTIONS

YJW participated in the animal experiment, histological experiment, experimental design, acquisition of data, data analysis and interpretation and manuscript writing. GH, YXS and HT acquired the experimental data of the immunostaining, Western blot and qRT‐PCR. MZ, XK and JTL joined the experimental design and manuscript revision. MZ, MYY and MDM contributed to the experimental design. XTB, FL and KL modified grammar and polished the manuscript. WC, BHZ and JQZ took part in the conception and design. KLT conducted the conception, design and manuscript writing. All authors read and approved the final manuscript.

## Data Availability

The authors declare that the data supporting the findings of this study are available within the article.
